# Association between low body mass index and increased 28-day mortality of severe sepsis in Japanese cohorts

**DOI:** 10.1038/s41598-020-80284-3

**Published:** 2021-01-15

**Authors:** Takehiko Oami, Satoshi Karasawa, Tadanaga Shimada, Taka-aki Nakada, Toshikazu Abe, Hiroshi Ogura, Atsushi Shiraishi, Shigeki Kushimoto, Daizoh Saitoh, Seitaro Fujishima, Toshihiko Mayumi, Yasukazu Shiino, Takehiko Tarui, Toru Hifumi, Yasuhiro Otomo, Kohji Okamoto, Yutaka Umemura, Joji Kotani, Yuichiro Sakamoto, Junichi Sasaki, Shin-ichiro Shiraishi, Kiyotsugu Takuma, Ryosuke Tsuruta, Akiyoshi Hagiwara, Kazuma Yamakawa, Tomohiko Masuno, Naoshi Takeyama, Norio Yamashita, Hiroto Ikeda, Masashi Ueyama, Satoshi Fujimi, Satoshi Gando, JAAM FORECAST Group, Osamu Tasaki, Yasumitsu Mizobata, Hiraku Funakoshi, Toshiro Okuyama, Iwao Yamashita, Toshio Kanai, Yasuo Yamada, Mayuki Aibiki, Keiji Sato, Susumu Yamashita, Susumu Yamashita, Kenichi Yoshida, Shunji Kasaoka, Akihide Kon, Hiroshi Rinka, Hiroshi Kato, Hiroshi Okudera, Eichi Narimatsu, Toshifumi Fujiwara, Manabu Sugita, Yasuo Shichinohe, Hajime Nakae, Ryouji Iiduka, Mitsunobu Nakamura, Yuji Murata, Yoshitake Sato, Hiroyasu Ishikura, Yasuhiro Myojo, Yasuyuki Tsujita, Kosaku Kinoshita, Hiroyuki Yamaguchi, Toshihiro Sakurai, Satoru Miyatake, Takao Saotome, Susumu Yasuda, Yasuaki Mizushima

**Affiliations:** 1 Department of Emergency and Critical Care Medicine, Chiba University Graduate School of Medicine, 1-8-1 Inohana, Chuo, Chiba 260-8677 Japan; 2 Department of General Medicine, Juntendo University, Tokyo, Japan; 3 Health Services Research and Development Center, University of Tsukuba, Tsukuba, Japan; 4 Department of Traumatology and Acute Critical Medicine, Osaka University Graduate School of Medicine, Osaka, Japan; 5 Emergency and Trauma Center, Kameda Medical Center, Kameda, Japan; 6 Division of Emergency and Critical Care Medicine, Tohoku University Graduate School of Medicine, Sendai, Japan; 7 Division of Traumatology, Research Institute, National Defense Medical College, Tokorozawa, Japan; 8 Center for General Medicine Education, Keio University School of Medicine, Tokyo, Japan; 9 Department of Emergency Medicine, School of Medicine, University of Occupational and Environmental Health, Kitakyushu, Japan; 10 Department of Acute Medicine, Kawasaki Medical School, Kurashiki, Japan; 11 Department of Trauma and Critical Care Medicine, Kyorin University School of Medicine, Mitaka, Japan; 12 Department of Emergency and Critical Care Medicine, St. Luke’s International Hospital, Tokyo, Japan; 13 Trauma and Acute Critical Care Center, Medical Hospital, Tokyo Medical and Dental University, Tokyo, Japan; 14 Department of Surgery, Center for Gastroenterology and Liver Disease, Kitakyushu City Yahata Hospital, Kitakyushu, Japan; 15 Division of Disaster and Emergency Medicine, Department of Surgery Related, Kobe University Graduate School of Medicine, Kobe, Japan; 16 Emergency and Critical Care Medicine, Saga University Hospital, Saga, Japan; 17 Department of Emergency and Critical Care Medicine, Keio University School of Medicine, Tokyo, Japan; 18 Department of Emergency and Critical Care Medicine, Aizu Chuo Hospital, Aizuwakamatsu, Japan; 19 Emergency and Critical Care Center, Kawasaki Municipal Kawasaki Hospital, Kawasaki, Japan; 20 Advanced Medical Emergency and Critical Care Center, Yamaguchi University Hospital, Ube, Japan; 21 Department of Emergency Medicine, Niizashiki Chuo General Hospital, Niiza, Japan; 22 Division of Trauma and Surgical Critical Care, Osaka General Medical Center, Osaka, Japan; 23 Department of Emergency and Critical Care Medicine, Nippon Medical School, Tokyo, Japan; 24 Advanced Critical Care Center, Aichi Medical University Hospital, Nagakute, Japan; 25 Advanced Emergency Medical Service Center, Kurume University Hospital, Fukuoka, Japan; 26 Department of Emergency Medicine, Teikyo University School of Medicine, Tokyo, Japan; 27 Department of Trauma, Critical Care Medicine, and Burn Center, Japan Community Healthcare Organization Chukyo Hospital, Nagoya, Japan; 28 Division of Acute and Critical Care Medicine, Hokkaido University Graduate School of Medicine, Sapporo, Japan; 29 Acute and Critical Care Center, Department of Acute and Critical Care Medicine, Sapporo Higashi Tokushukai Hospital, Sapporo, Japan; 30 Japan Association for Acute Medicine, Tokyo, Japan; 31 Nagasaki University Hospital, Nagasaki, Japan; 32 Osaka City University Hospital, Osaka, Japan; 33 Tokyobay Urayasu Ichikawa Medical Center, Urayasu, Japan; 34 Aso Iizuka Hospital, Fukuoka, Japan; 35 Tomei Atsugi Hospital, Atsugi, Japan; 36 Hiratsuka City Hospital, Hiratsuka, Japan; 37 National Hospital Organization Sendai Medical Center, Sendai, Japan; 38 Ehime University Hospital, Toon, Japan; 39 Okayama University Hospital, Okayama, Japan; 40 Tokuyama Central Hospital, Shunan, Japan; 41 Fukuyama City Hospital, Fukuyama, Japan; 42 JA Hiroshima General Hospital, Hiroshima, Japan; 43 Kumamoto University Hospital, Kumamoto, Japan; 44 Hachinohe City Hospital, Hachinohe, Japan; 45 Osaka City General Hospital, Osaka, Japan; 46 National Hospital Organization Disaster Medical Center, Nagoya, Japan; 47 University of Toyama, Toyama, Japan; 48 Sapporo Medical University, Sapporo, Japan; 49 Okayama Saiseikai General Hospital, Okayama, Japan; 50 Juntendo University Nerima Hospital, Tokyo, Japan; 51 National Hospital Organization Hokkaido Medical Center, Sapporo, Japan; 52 Akita University Hospital, Akita, Japan; 53 Japanese Red Cross Society Kyoto Daini Hospital, Kyoto, Japan; 54 Maebashi Red Cross Hospital, Maebashi, Japan; 55 Sendai City Hospital, Sendai, Japan; 56 Subaru Health Insurance Society Ota Memorial Hospital, Ota, Japan; 57 Fukuoka University Hospital, Fukuoka, Japan; 58 Ishikawa Prefectural Central Hospital, Kanazawa, Japan; 59 Shiga University of Medical Science, Shiga, Japan; 60 Nihon University School of Medicine, Tokyo, Japan; 61 Seirei Yokohama General Hospital, Yokohama, Japan; 62 National Hospital Organization Kumamoto Medical Center, Kumamoto, Japan; 63 Saiseikai Utsunomiya Hospital, Utsunomiya, Japan; 64 National Hospital Organization Higashi-Ohmi General Medical Center, Higashi-ohmi, Japan; 65 National Hospital Organization Mito Medical Center, Ibaraki, Japan; 66 Rinku General Medical Center, Izumisano, Japan

**Keywords:** Infectious diseases, Metabolic disorders, Infection, Inflammation

## Abstract

Current research regarding the association between body mass index (BMI) and altered clinical outcomes of sepsis in Asian populations is insufficient. We investigated the association between BMI and clinical outcomes using two Japanese cohorts of severe sepsis (derivation cohort, Chiba University Hospital, n = 614; validation cohort, multicenter cohort, n = 1561). Participants were categorized into the underweight (BMI < 18.5) and non-underweight (BMI ≥ 18.5) groups. The primary outcome was 28-day mortality. Univariate analysis of the derivation cohort indicated increased 28-day mortality trend in the underweight group compared to the non-underweight group (underweight 24.4% [20/82 cases] vs. non-underweight 16.0% [85/532 cases]; *p* = 0.060). In the primary analysis, multivariate analysis adjusted for baseline imbalance revealed that patients in the underweight group had a significantly increased 28-day mortality compared to those in the non-underweight group (*p* = 0.031, adjusted odds ratio [OR] 1.91, 95% confidence interval [CI] 1.06–3.46). In a repeated analysis using a multicenter validation cohort (underweight n = 343, non-underweight n = 1218), patients in the underweight group had a significantly increased 28-day mortality compared to those in the non-underweight group (*p* = 0.045, OR 1.40, 95% CI 1.00–1.97). In conclusion, patients with a BMI < 18.5 had a significantly increased 28-day mortality compared to those with a BMI ≥ 18.5 in Japanese cohorts with severe sepsis.

## Introduction

Sepsis is defined as life-threatening organ dysfunction caused by a dysregulated host response to infection^1^; the excessive spillover of humoral mediators, including interleukin (IL)-6, into the systemic circulation is a well-known component of the dysregulated response^2^. Body mass index (BMI), which is a measure of body fat, is associated with an altered inflammatory response^3^. BMI is a non-invasively measurable physical characteristic and clinically practical variable; therefore, greater understanding of the relationship between BMI and altered clinical outcomes may contribute to improvements in basic sepsis care^4^.

A recent systematic review found eight studies investigating whether BMI was associated with altered outcome of sepsis; however, all studies were conducted in either Europe or North America^5^. The systematic review concluded that patients with an increased BMI improved survival following sepsis^4,6^. By contrast, patients with a low BMI significantly increased blood IL-6 levels compared to those with a high BMI in North America^3^. However, studies regarding BMI in sepsis in Asian populations are limited; after the publication of the systematic review, a single-center study of sepsis with a small sample size in China reported that patients with a low BMI had increased mortality^7^. Non-obese patients were more common in Asian countries than in Europe and North America^8–10^.

Thus, we tested the hypothesis that patients with a lower BMI have worse clinical outcomes through an altered inflammatory response using large Japanese cohorts of severe sepsis. The primary outcome was 28-day mortality, and blood IL-6 levels were measured in the derivation cohort.

## Methods

### Study setting and patients

This observational study deployed the following severe sepsis cohorts. The methods were conducted in accordance with the Declaration of Helsinki and relevant guidelines.

### Derivation cohort: a single-center cohort

Patients admitted to the intensive care unit (ICU) at Chiba University Hospital, Japan, between October 2012 and May 2019 were retrospectively screened, and patients with severe sepsis were assessed for eligibility^11^. Patients who had missing data regarding BMI and mortality were excluded.

### Validation cohort: multicenter cohorts

The validation cohort consisted of Japanese Association for Acute Medicine Sepsis Registry (JAAMSR) and Focused Outcomes Research in Emergency Care in Acute Respiratory Distress Syndrome, Sepsis, and Trauma (FORECAST) cohorts. These two cohorts were multicenter, prospective, observational studies conducted by the Japanese Association for Acute Medicine (JAAM) without an overlapping duration for patient enrollment. JAAMSR recruited patients with severe sepsis from 15 ICUs in Japan between June 2010 and May 2011. FORECAST, which followed JAAMSR and was conducted by the JAAM, enrolled study participants with severe sepsis from 59 ICUs in Japan between January 2016 and March 2017. We made the decision to combine the two cohorts with the aim of strengthening the robustness. Because our institution had participated in FORECAST, the population that overlapped with the derivation cohort was removed from the validation cohort.

### Data collection and definition

We chose our normal BMI range in accordance with the World Health Organization (WHO) classification (18.5 ≤ BMI < 25.0). The WHO classification has two high BMI categories (25.0 ≤ BMI < 30.0 and BMI ≥ 30.0). However, due to the small sample size of the highest BMI category (BMI ≥ 30.0, 7.3% in the derivation cohort), we combined the two high BMI categories. We first screened for differences in mortality between the abnormal BMI (underweight, BMI < 18.5 or overweight, BMI ≥ 25.0) and normal (18.5 ≤ BMI < 25.0) groups in the derivation cohort. Significant discovery results were tested for replication and generalizability in a multicenter validation cohort.

Blood IL-6 levels in the derivation cohort were rapidly measured after blood sample collections on days 1, 2, and 3 at the clinical laboratory in Chiba University Hospital using rapid measurement systems (IL-6, Roche Diagnostics, Tokyo, Japan)^12^. Raw data were converted into a logarithmic scale for analysis.

Severe sepsis and septic shock were defined according to the Sepsis-2 criteria^11^. All patients received treatment according to the international guidelines for the management of severe sepsis and septic shock^13,14^.

### Statistical analysis

Data are presented as medians (quartiles). Categorical data were analyzed using the Pearson’s chi-square test. The Mann–Whitney U test or Kruskal–Wallis test was used for unpaired comparisons depending on the number of groups.

Multivariate logistic regression was used to analyze 28-day mortality by the BMI category. We selected this approach to adjust for potential baseline imbalances, including age, sex, the Sequential Organ Failure Assessment (SOFA) score, and site of infection. We compared blood IL-6 levels measured on days 1, 2 and 3 between the BMI categories using a generalized estimating equation.

Statistical significance was determined by a two-tailed *p* value < 0.05. Data were analyzed using SPSS software version 24.0 (IBM Corporation, Armonk, NY, USA) and GraphPad Prism 6 (GraphPad Software, San Diego, CA, USA).

### Ethical approval and consent to participate

The institutional review board at Chiba University Graduate School of Medicine approved this study and waived the need for written informed consent from subjects or their legal surrogates.

## Results

### Baseline patient characteristics

Of the 785 patients treated during the study period, 614 were included in this analysis (Supplementary Fig. S1 online). Participants were categorized by BMI into the following groups: underweight (n = 82), normal weight (n = 350), and overweight (n = 182). No significant differences were observed in baseline characteristics, except increased probability of diabetes mellitus, higher white blood cell counts, and serum creatinine levels in the overweight group (Supplementary Table S1 online).

### Primary outcome in the derivation cohort

The 28-day mortality rate was 24.4%, 15.7%, and 16.5% in the underweight, normal, and overweight groups, respectively. According to the screening analysis, we chose the potential threshold of BMI 18.5 and further analyzed between the underweight (BMI < 18.5) and non-underweight (BMI ≥ 18.5) groups (Table 1). Univariate analysis revealed a non-significant trend of increased 28-day mortality in the underweight group (24.4%) compared to the non-underweight group (16.0%) (*p* = 0.060).

**Table 1:** Baseline characteristics in the derivation cohort.

	Underweight (BMI < 18.5) (n = 82)	Non-underweight (BMI ≥ 18.5) (n = 532)	*p* value
Age, yr	70 (57–77)	69 (61–76)	0.62
Male sex, n (%)	51 (62.2)	355 (66.7)	0.41
Body mass index	17.3 (16.0–17.8)	23.3 (20.9–26.3)	< 0.0001
**Site of infection, n (%)**			0.33
Lung	39 (47.6)	207 (38.9)	
Intra-abdominal	24 (29.3)	162 (30.5)	
Urinary tract	8 (9.8)	44 (8.3)	
Skin and soft tissue	4 (4.9)	56 (10.5)	
Others	7 (8.5)	63 (11.8)	
**Comorbidity, n (%)**			
Diabetes mellitus	12 (14.6)	152 (28.6)	0.008
Stroke	5 (6.1)	17 (3.2)	0.18
Malignancy	29 (35.4)	153 (28.8)	0.22
Heart failure	10 (12.2)	48 (9.0)	0.36
Chronic kidney disease	8 (9.8)	40 (7.5)	0.48
Liver disease	7 (8.5)	33 (6.2)	0.42
Chronic lung disease	8 (9.8)	25 (4.7)	0.059
Septic shock, n (%)	41 (50.0)	252 (47.4)	0.65
SOFA score	11 (9–14)	12 (9–15)	0.38
APACHE II score	29 (24–36)	29 (23–36)	0.72
Mechanical ventilation, n (%)	38 (46.3)	226 (42.5)	0.51
Catecholamine, n (%)	51 (62.2)	329 (61.8)	0.95
**Laboratory data on day 1**			
White blood cell (× 10^3^/mm^3^)	8.5 (3.4–14.0)	11.5 (5.9–17.0)	0.012
Creatinine (mg/dL)	1.3 (0.7–2.2)	1.4 (0.9–2.6)	0.10
Lactate (mmol/L)	2.5 (1.5–5.7)	2.4 (1.4–4.8)	0.49

Data are presented as median (quartile).

*BMI* body mass index, *SOFA* sequential organ failure assessment, *APACHE* acute physiology and chronic health evaluation.

Multivariate logistic regression analysis to adjust for potential baseline imbalances including age, male sex, the SOFA score, and site of infection indicated that patients with severe sepsis in the underweight group had a significantly increased 28-day mortality compared to those in the non-underweight group (*p* = 0.031, adjusted odds ratio [OR] 1.91, 95% confidence interval [CI] 1.06–3.46) (Table 2A).

**Table 2:** Multivariate logistic regression analysis of 28-day mortality.

Variable	Odds ratio	95% CI	*p* value
**A. Derivation cohort**			
Age-per year	1.02	1.00–1.04	0.008
Male sex	0.82	0.50–1.34	0.43
SOFA	1.18	1.11–1.25	< 0.0001
**Site of infection**			
Lung	1.00	Reference	
Intra-abdominal	0.45	0.26–0.80	0.006
Urinary tract	0.50	0.20–1.26	0.14
Skin and soft tissue	0.48	0.19–1.22	0.12
Others	1.04	0.52–2.09	0.89
Underweight (BMI < 18.5)	1.91	1.06–3.46	0.031
**B. Validation cohort**			
Age-per year	1.02	1.01–1.03	< 0.0001
Male sex	1.07	0.79–1.44	0.64
SOFA	1.22	1.17–1.27	< 0.0001
**Site of infection**			
Lung	1.00	Reference	
Intra-abdominal	0.72	0.50–1.05	0.092
Urinary tract	0.45	0.28–0.74	0.002
Skin and soft tissue	0.84	0.51–1.40	0.51
Others	1.27	0.83–1.94	0.26
**Type of cohort**			
JAAMSR	1.00	Reference	
FORECAST	0.72	0.54–0.96	0.26
Underweight (BMI < 18.5)	1.40	1.00–1.97	0.045
**C. Combined cohorts**			
Age-per year	1.02	1.01–1.03	< 0.0001
Male sex	1.00	0.78–1.29	0.64
SOFA	1.20	1.16–1.24	< 0.0001
**Site of infection**			
Lung	1.00	Reference	
Intra-abdominal	0.61	0.45–0.84	0.002
Urinary tract	0.45	0.29–0.69	< 0.0001
Skin and soft tissue	0.73	0.47–1.13	0.16
Others	1.19	0.83–1.70	0.34
**Type of cohort**			
JAAMSR	1.00	Reference	
Single center cohort	0.42	0.30–0.59	< 0.0001
FORECAST	0.77	0.58–1.03	0.083
Underweight (BMI < 18.5)	1.50	1.12–2.00	0.006

*CI* confidence interval, *SOFA* sequential organ failure assessment, *BMI* body mass index.

### Primary outcome in the validation cohort

To validate the increased mortality of the underweight group, we deployed a validation cohort enrolling 1561 patients (underweight n = 343, non-underweight n = 1218) (Supplementary Fig. S2 online). There were significant differences in age, site of infection, proportion of patients with diabetes mellitus and chronic lung disease, white blood cell count, and serum creatinine levels according to BMI classifications (Supplementary Table S2 online). A repeated multivariate analysis indicated that patients with severe sepsis in the underweight group had a significantly increased 28-day mortality compared to those in the non-underweight group (*p* = 0.045, adjusted OR 1.40, 95% CI 1.00–1.97) (Table 2B). To rule out the bias due to the comorbidities, we performed another logistic regression model using Charlson co-morbidities index with the data set of FORECAST study. As a result, the underweight group showed consistent worse outcome after correction with comorbidities.

### Primary outcome in the combined cohorts

To investigate the accuracy of the findings in the derivation and validation cohorts, we combined the two cohorts enrolling 2175 patients (underweight n = 425, non-underweight n = 1750). There were significant differences in age, site of infection, proportion of patients with diabetes mellitus, stroke, and chronic lung disease, white blood cell count, and serum creatinine levels between the underweight and non-underweight group (Supplementary Fig. S3 online). The underweight group remained a significant predictor for 28-day mortality in the logistic regression model (*p* = 0.006, adjusted OR 1.50, 95% CI 1.12–2.00) (Table 2C).

### IL-6 analysis in the derivation cohort

**Figure 1:**
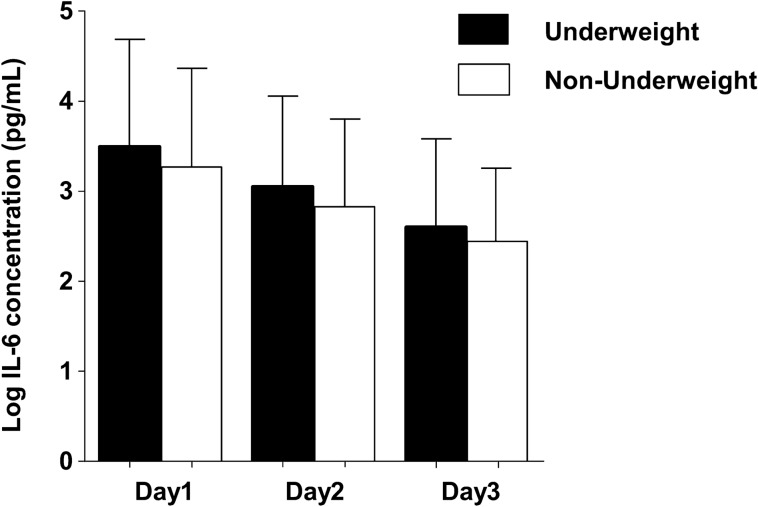
Comparison of blood IL-6 levels between the underweight (BMI < 18.5) and non-underweight (BMI ≥ 18.5) group at days 1, 2, and 3. There was a non-significant trend of increased blood IL-6 levels in the underweight group compared to the non-underweight group in a generalized estimating equation using log-converted serum IL-6 concentration at days 1, 2, and 3 (*p* = 0.088, adjusted OR 1.24, 95% CI 0.96–1.60). *IL* interleukin, *BMI* body mass index.

Data regarding blood IL-6 levels were available for 413 of the 614 study subjects. There was a non-significant trend of increased blood IL-6 levels in the underweight group compared to the non-underweight group, as determined through a generalized estimating equation using log-converted blood IL-6 levels from days 1, 2, and 3 (*p* = 0.088, adjusted OR 1.24, 95% CI 0.96–1.60) (Fig. 1). To evaluate the predictive accuracy of the logistic regression model after adjustment with blood IL-6 levels, we performed another analysis predicting 28-day mortality. While the underweight group showed a consistent significance as an independent predictor for 28-day mortality with either of IL-6 levels, only IL-6 levels at day3, but not IL-6 levels at day1 and day2, was a significant variable for predicting the outcome during sepsis (Supplementary Table S4 online).

## Discussion

The present study of severe sepsis, using two large Japanese cohorts, found that patients with a BMI < 18.5 had an increased 28-day mortality. There was a trend of increased blood IL-6 levels in patients with a BMI < 18.5 during the initial 3 days.

Over the years, whether BMI was associated with altered mortality of patients with severe sepsis has been rarely reported in Asian population; we only found one single-center study with a small sample size (sepsis n = 178) conducted in a Chinese population. In the Chinese study, patients with a BMI < 18.5 had the highest 90-day mortality (66.7%) compared to the other subgroups with BMI ≥ 18.5 (18.2–48.0%)^7^. In accordance with these findings, the present study that enrolled 2175 patients with severe sepsis in total verified the increased mortality in patients with a BMI < 18.5. While the recent meta-analysis of BMI studies in Europe, North America, and Australia highlighted the benefit of obesity in sepsis^15^, two sepsis studies in North America revealed patients with a low BMI had increased mortality, which were in line with the results of this study^16,17^. Notably, the underweight group only accounted for 4.9–6% of the North American study, whereas our two cohorts included more than three times (19%) the percentage of patients with low BMI^16,17^. Therefore, our study is potentially more robust with regard to the conclusions derived from the data.

Differences in physical characteristics between the Asian and Western population, including body fat and muscle mass, have been widely recognized^8,9^. As such, the average BMI of 22.1 in the present study was lower than the BMI reported in previous European and North American studies (range 25.1–26.1)^18,19^ but similar to the median BMI of 22.5 that was previously reported in critically ill Japanese patients^20^. A single-center Japanese study including all critically ill patients (n = 1,616, sepsis fraction unknown) revealed that patients with a BMI < 18.5 had a significantly increased mortality compared to those with a BMI ≥ 18.5^20^, thus supporting the use of the BMI < 18.5 threshold for distinguishing low BMI groups in Asian populations^10^.

Plausible mechanisms postulated by bench studies could strengthen the relationship between low BMI populations and worse outcomes following sepsis. Lipoproteins and adipose tissue have a protective effect on the survival of sepsis patients through binding pathogen lipids and sequestering lipopolysaccharide (LPS), which could decrease systemic inflammation^21,22^. As proof of these interactions, our study highlighted the trend of higher blood IL-6 levels in patients with a lower BMI. An elevation of blood IL-6 levels, used for classifying severity of sepsis^23–25^, reinforces the plausibility of poor outcomes in patients with a low BMI. Furthermore, in accordance with our results, Wacharasint et al. also found that in Canadian population, patients with a BMI < 25 had worse outcomes and higher blood IL-6 levels^3^.

This study has limitations that need to be addressed. First, we analyzed data retrospectively. Second, we used BMI as a surrogate value to assess the percentage of body fat, but this might not be accurate for the evaluation of metabolic status without more detailed information. Therefore, adding other measurements, such as muscle volume or lipid markers, would be greatly beneficial to further understand the effect of metabolism on clinical outcomes following sepsis.

## Conclusions

In Japanese cohorts of severe sepsis, patients with a BMI < 18.5 had a significantly increased 28-day mortality compared to those with a BMI ≥ 18.5.

## Supplementary Information

Supplementary Information.

## Data Availability

The datasets used and analyzed during our study are available from the corresponding author upon reasonable request.
