# Supplementary material for: Association between low body mass index and increased 28-day mortality of severe sepsis in Japanese cohorts
Source: Sci Rep. 2021 Jan 15;11:1615. doi: 10.1038/s41598-020-80284-3 (PMC7810989; doi:10.1038/s41598-020-80284-3)
Supplement: Supplementary file 1 — Supplementary Information. [file 41598_2020_80284_MOESM1_ESM.doc]

**Association between low body mass index and increased 28-day mortality of severe sepsis in Japanese cohorts**

**Authors:** Takehiko Oami, Satoshi Karasawa, Tadanaga Shimada, Taka-aki Nakada, Toshikazu Abe, Hiroshi Ogura, Atsushi Shiraishi, Shigeki Kushimoto, Daizoh Saitoh, Seitaro Fujishima, Toshihiko Mayumi, Yasukazu Shiino, Takehiko Tarui, Toru Hifumi, Yasuhiro Otomo, Kohji Okamoto, Yutaka Umemura, Joji Kotani, Yuichiro Sakamoto, Junichi Sasaki, Shin-ichiro Shiraishi, Kiyotsugu Takuma, Ryosuke Tsuruta, Akiyoshi Hagiwara, Kazuma Yamakawa, Tomohiko Masuno, Naoshi Takeyama, Norio Yamashita, Hiroto Ikeda, Masashi Ueyama, Satoshi Fujimi, Satoshi Gando, on behalf of the JAAM FORECAST Group

***Online data supplement***

**Table S1.** Baseline characteristics in the derivation cohort among underweight, normal weight, and overweight group

**Table S2.** Baseline characteristics in the validation cohort

**Table S3.** Baseline characteristics in the combined cohorts

**Table S4.** Multivariate logistic regression analysis of 28-day mortality with blood IL-6 levels

**Figure S1.** Flowchart of study enrollment in the derivation cohort

**Figure S2.** Flowchart of study enrollment in the validation cohort

**Table S1. Baseline characteristics in the derivation cohort**

|  | Underweight  (BMI<18.5)  (n = 82) | Normal weight  (18.5≤BMI<25.0)  (n = 350) | Overweight (BMI≥25.0)  (n = 182) | p*-*value |
| --- | --- | --- | --- | --- |
| Age, yr | 70 (57–77) | 70 (62–76) | 68 (55-74) | 0.063 |
| Male sex, n (%) | 51 (62.2) | 238 (68.0) | 117 (64.3) | 0.49 |
| Body mass index | 17.3 (16.0–17.8) | 21.7 (20.3–23.2) | 27.5 (26.2–29.6) | <0.0001 |
| Site of infection, n (%) |  |  |  | 0.061 |
| Lung | 39 (47.6) | 151 (43.1) | 56 (30.8) |  |
| Intra-abdominal | 24 (29.3) | 105 (30.0) | 57 (31.3) |  |
| Urinary tract | 8 (9.8) | 26 (7.4) | 18 (9.9) |  |
| Skin and soft tissue | 4 (4.9) | 30 (8.6) | 26 (14.3) |  |
| Others | 7 (8.5) | 38 (10.9) | 25 (13.7) |  |
| Comorbidity, n (%) |  |  |  |  |
| Diabetes mellitus | 12 (14.6) | 89 (25.4) | 63 (34.6) | 0.002 |
| Stroke | 5 (6.1) | 12 (3.4) | 5 (2.7) | 0.38 |
| Malignancy | 29 (35.4) | 103 (29.4) | 50 (27.5) | 0.42 |
| Heart failure | 10 (12.2) | 29 (8.3) | 19 (10.4) | 0.47 |
| Chronic kidney disease | 8 (9.8) | 31 (8.9) | 9 (4.9) | 0.21 |
| Liver disease | 7 (8.5) | 19 (5.4) | 14 (7.7) | 0.44 |
| Chronic lung disease | 8 (9.8) | 17 (4.9) | 8 (4.4) | 0.16 |
| Septic shock, n (%) | 41 (50.0) | 166 (47.4) | 86 (47.3) | 0.90 |
| SOFA score | 11 (9–14) | 12 (9–15) | 12 (9–15) | 0.59 |
| APACHE II score | 29 (24–36) | 29 (23–36) | 29 (24–36) | 0.84 |
| Mechanical ventilation, n (%) | 38 (46.3) | 143 (40.9) | 83 (45.6) | 0.46 |
| Catecholamine, n (%) | 51 (62.2) | 214 (61.1) | 115 (63.2) | 0.89 |
| Laboratory data on day 1 |  |  |  |  |
| White blood cell (×10^3^/mm^3^) | 8.5 (3.4–14.0) | 11.5 (5.0–17.0) | 12.0 (6.5–16.5) | 0.043 |
| Creatinine (mg/dL) | 1.3 (0.7–2.2) | 1.3 (0.8–2.2) | 1.8 (1.0–3.1) | <0.0001 |
| Lactate (mmol/L) | 2.5 (1.5–5.7) | 2.6 (1.5–5.0) | 2.1 (1.3–4.8) | 0.31 |

Data are presented as median (quartile).

BMI, body mass index; SOFA, Sequential Organ Failure Assessment; APACHE, Acute Physiology and Chronic Health Evaluation.

**Table S2.** Baseline characteristics in the validation cohort

|  | Underweight (BMI<18.5)  (n = 343) | Non-underweight (BMI≥18.5)  (n = 1218) | p*-*value |
| --- | --- | --- | --- |
| Age, yr | 75 (64–83) | 72 (62–81) | 0.007 |
| Male sex, n (%) | 199 (58.0) | 752 (61.7) | 0.21 |
| Body mass index | 16.8 (15.5–17.7) | 22.7 (20.5–25.4) | <0.0001 |
| Site of infection, n (%) |  |  | <0.0001 |
| Lung | 141 (41.1) | 377 (31.0) |  |
| Intra-abdominal | 83 (24.2) | 297 (24.4) |  |
| Urinary tract | 63 (18.4) | 206 (16.9) |  |
| Skin and soft tissue | 20 (5.8) | 156 (12.8) |  |
| Others | 36 (10.5) | 182(14.9) |  |
| Comorbidity, n (%) |  |  |  |
| Diabetes mellitus | 58 (16.9) | 283 (23.2) | 0.012 |
| Stroke | 52 (15.2) | 144 (11.8) | 0.099 |
| Malignancy | 45 (13.1) | 167 (13.7) | 0.77 |
| Heart failure | 37 (10.8) | 110 (9.0) | 0.32 |
| Chronic kidney disease | 28 (8.2) | 81 (6.7) | 0.33 |
| Liver disease | 21 (6.1) | 64 (5.3) | 0.53 |
| Chronic lung disease | 26 (7.6) | 56 (4.6) | 0.029 |
| Septic shock, n (%) | 202 (58.9) | 698 (57.3) | 0.60 |
| SOFA score | 9 (6–11) | 9 (6-11) | 0.96 |
| APACHE II score | 23 (18–30) | 22 (17–29) | 0.053 |
| Mechanical ventilation, n (%) | 157 (45.8) | 597 (49.0) | 0.50 |
| Catecholamine, n (%) | 211 (61.5) | 740 (60.8) | 0.59 |
| Laboratory data on day 1 |  |  |  |
| White blood cell (×10^3^/mm^3^) | 11.4 (4.9-17.2) | 12.0 (6.3-18.4) | 0.045 |
| Creatinine (mg/dL) | 1.3 (0.7-2.4) | 1.6 (0.9-2.7) | <0.0001 |
| Lactate (mmol/L) | 3.2 (2.0–5.5) | 3.1 (1.9–5.3) | 0.18 |

Data are presented as median (quartile).

BMI, body mass index; SOFA, Sequential Organ Failure Assessment; APACHE, Acute Physiology and Chronic Health Evaluation.

**Table S3.** Baseline characteristics in the combined cohorts

|  | Underweight (BMI<18.5)  (n = 425) | Non-underweight (BMI≥18.5)  (n = 1750) | p*-*value |
| --- | --- | --- | --- |
| Age, yr | 74 (63–82) | 71 (62–79) | 0.001 |
| Male sex, n (%) | 25.0 (58.8) | 1107 (63.3) | 0.094 |
| Body mass index | 16.9 (15.6–17.7) | 22.9 (20.6–25.6) | <0.0001 |
| Site of infection, n (%) |  |  | <0.0001 |
| Lung | 180 (42.4) | 584 (33.4) |  |
| Intra-abdominal | 107 (25.2) | 459 (26.2) |  |
| Urinary tract | 71 (16.7) | 250 (14.3) |  |
| Skin and soft tissue | 24 (5.6) | 212 (12.1) |  |
| Others | 43 (10.1) | 245 (14.0) |  |
| Comorbidity, n (%) |  |  |  |
| Diabetes mellitus | 70 (16.5) | 435 (24.9) | <0.0001 |
| Stroke | 57 (13.4) | 161 (9.2) | 0.012 |
| Malignancy | 74 (17.4) | 320 (18.3) | 0.72 |
| Heart failure | 47 (11.1) | 158 (9.0) | 0.19 |
| Chronic kidney disease | 36 (8.5) | 121 (6.9) | 0.29 |
| Liver disease | 28 (6.6) | 97 (5.5) | 0.41 |
| Chronic lung disease | 34 (8.0) | 81 (4.6) | 0.008 |
| Septic shock, n (%) | 224 (52.7) | 852 (48.7) | 0.14 |
| SOFA score | 9 (6–12) | 10 (6-13) | 0.071 |
| APACHE II score | 25 (18–31) | 24 (18–31) | 0.63 |
| Mechanical ventilation, n (%) | 195 (45.9) | 823 (47.0) | 0.70 |
| Catecholamine, n (%) | 242 (56.9) | 943 (53.9) | 0.27 |
| Laboratory data on day 1 |  |  |  |
| White blood cell (×10^3^/mm^3^) | 10.8 (4.4-16.7) | 11.8 (6.1-17.9) | 0.007 |
| Creatinine (mg/dL) | 1.3 (0.7-2.4) | 1.5 (0.9-2.7) | <0.0001 |
| Lactate (mmol/L) | 3.1 (1.9–5.6) | 2.9 (1.7–5.2) | 0.51 |

Data are presented as median (quartile).

BMI, body mass index; SOFA, Sequential Organ Failure Assessment; APACHE, Acute Physiology and Chronic Health Evaluation.

**Table S4.** **Multivariate logistic regression analysis of 28-day mortality with blood IL-6 levels**

1. **Regression model with blood IL-6 levels at day1**

| Variable | Odds ratio | 95% CI | p*-*value |
| --- | --- | --- | --- |
| Age -per year | 1.02 | 1.00-1.04 | 0.016 |
| Male sex | 1.07 | 0.58-1.98 | 0.80 |
| SOFA | 1.21 | 1.12-1.30 | <0.0001 |
| Site of infection |  |  |  |
| Lung | 1.00 | Reference |  |
| Intra-abdominal | 0.53 | 0.26-1.05 | 0.071 |
| Urinary tract | 0.42 | 0.10-1.66 | 0.22 |
| Skin and soft tissue | 0.50 | 0.17-1.43 | 0.20 |
| Others | 0.86 | 0.35-2.12 | 0.75 |
| Underweight (BMI<18.5) | 2.51 | 1.06-3.46 | 0.031 |
| Blood IL-6 levels at day1 | 0.88 | 0.67-1.16 | 0.38 |

1. **Regression model with blood IL-6 levels at day2**

| Variable | Odds ratio | 95% CI | p*-*value |
| --- | --- | --- | --- |
| Age -per year | 1.02 | 1.00-1.04 | 0.033 |
| Male sex | 0.97 | 0.54-1.74 | 0.92 |
| SOFA | 1.18 | 1.11-1.27 | <0.0001 |
| Site of infection |  |  |  |
| Lung | 1.00 | Reference |  |
| Intra-abdominal | 0.42 | 0.22-0.82 | 0.012 |
| Urinary tract | 0.45 | 0.11-1.74 | 0.25 |
| Skin and soft tissue | 0.45 | 0.15-1.28 | 0.13 |
| Others | 1.03 | 0.43-2.42 | 0.94 |
| Underweight (BMI<18.5) | 2.07 | 1.01-4.22 | 0.045 |
| Blood IL-6 levels at day2 | 1.14 | 0.86-1.53 | 0.34 |

1. **Regression model with blood IL-6 levels at day3**

| Variable | Odds ratio | 95% CI | p*-*value |
| --- | --- | --- | --- |
| Age -per year | 1.00 | 0.98-1.03 | 0.39 |
| Male sex | 1.19 | 0.63-2.22 | 0.58 |
| SOFA | 1.15 | 1.06-1.24 | <0.0001 |
| Site of infection |  |  |  |
| Lung | 1.00 | Reference |  |
| Intra-abdominal | 0.35 | 0.17-0.72 | 0.005 |
| Urinary tract | 0.48 | 0.12-1.89 | 0.29 |
| Skin and soft tissue | 0.35 | 0.11-1.14 | 0.082 |
| Others | 0.56 | 0.21-1.47 | 0.24 |
| Underweight (BMI<18.5) | 2.33 | 1.09-4.95 | 0.028 |
| Blood IL-6 levels at day3 | 1.44 | 1.00-2.09 | 0.049 |

CI, confidence interval; SOFA, Sequential Organ Failure Assessment; BMI, body mass index; IL, interleukin.

**Figure S1.** Flowchart of study enrollment in the derivation cohor
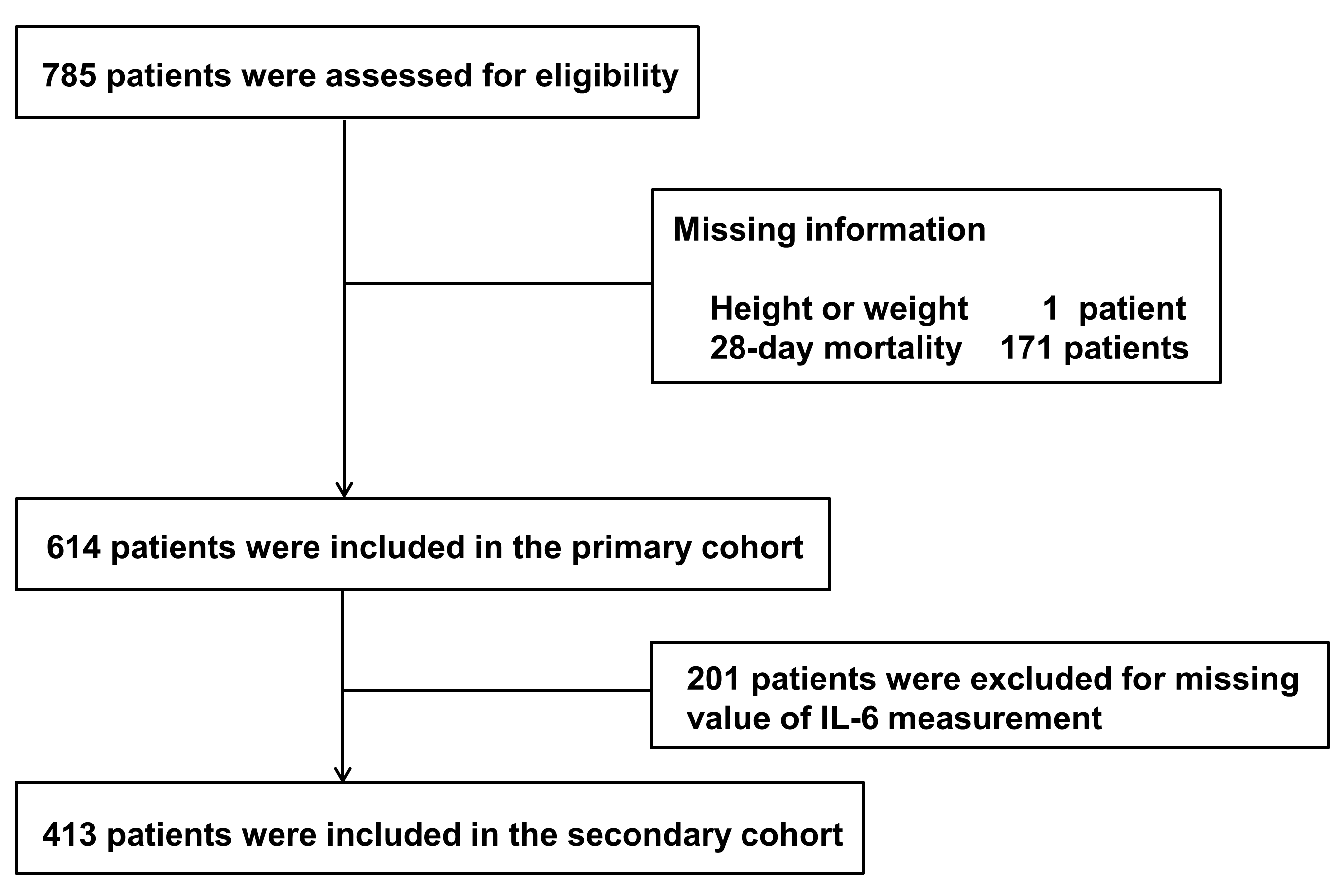
t

**Figure S2.** Flowchart of study enrollment in the validation cohort

**
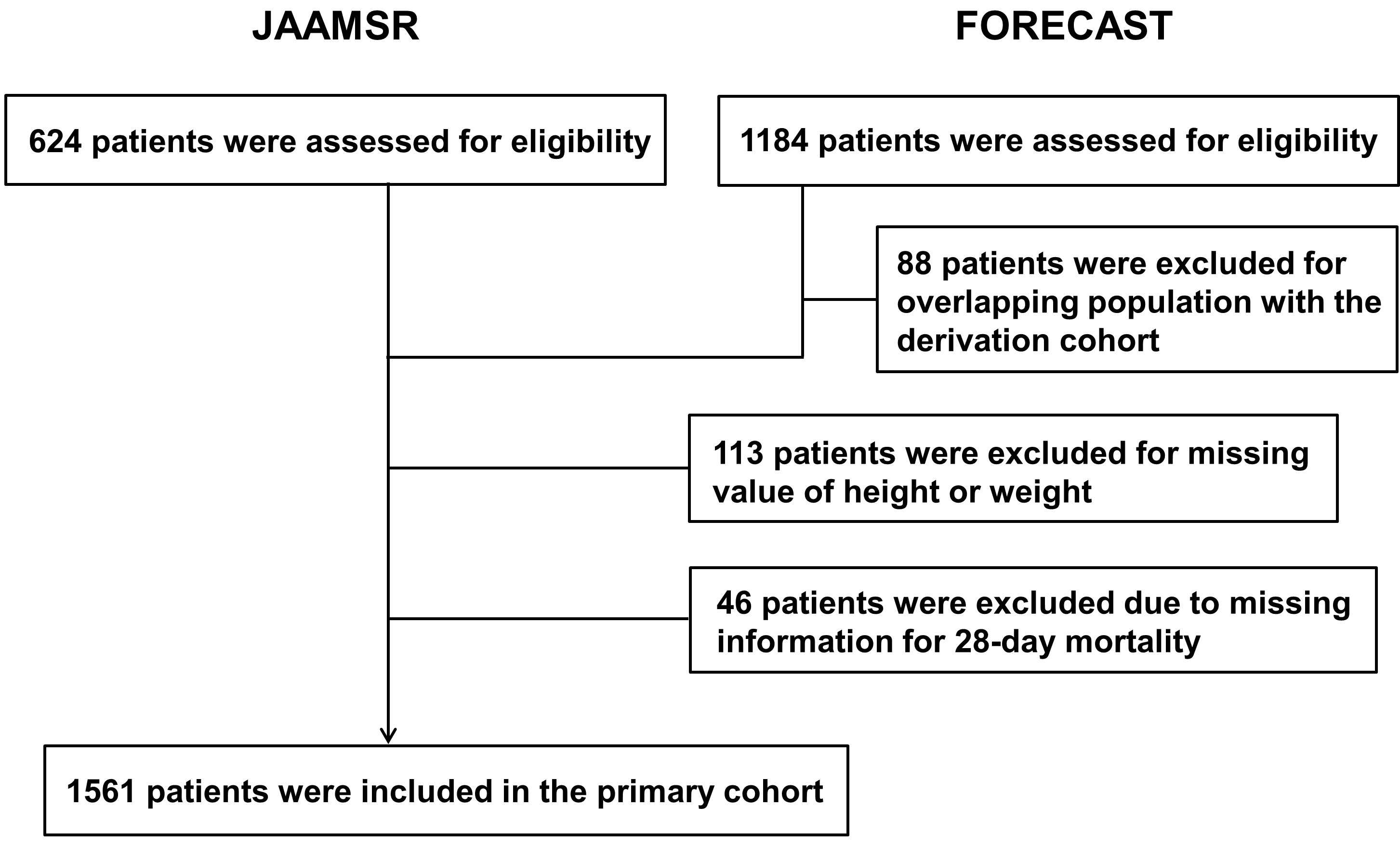
**
